# Using Online Screening in the General Population to Detect Participants at Clinical High-Risk for Psychosis

**DOI:** 10.1093/schbul/sby069

**Published:** 2018-06-08

**Authors:** Mhairi McDonald, Eleni Christoforidou, Nicola Van Rijsbergen, Ruchika Gajwani, Joachim Gross, Andrew I Gumley, Stephen M Lawrie, Matthias Schwannauer, Frauke Schultze-Lutter, Peter J Uhlhaas

**Affiliations:** 1 Institute of Neuroscience and Psychology, University of Glasgow, Glasgow, UK; 2 Institute of Health and Wellbeing, University of Glasgow, Glasgow, UK; 3 Institute for Biomagnetism and Biosignalanalysis, University of Muenster, Germany; 4 Department of Psychiatry, University of Edinburgh, Edinburgh, UK; 5 Department of Clinical Psychology, University of Edinburgh, Edinburgh, UK; 6 University Hospital of Child and Adolescent Psychiatry and Psychotherapy, University of Bern, Bern, Switzerland; 7 Department of Psychiatry and Psychotherapy, Medical Faculty, Heinrich Heine University, Düsseldorf, Germany

**Keywords:** clinical high-risk, psychosis, early intervention, web screening, basic symptoms

## Abstract

**Introduction:**

Identification of participants at clinical high-risk (CHR) for the development of psychosis is an important objective of current preventive efforts in mental health research. However, the utility of using web-based screening approaches to detect CHR participants at the population level has not been investigated.

**Methods:**

We tested a web-based screening approach to identify CHR individuals. Potential participants were invited to a website via e-mail invitations, flyers, and invitation letters involving both the general population and mental health services. Two thousand two hundred seventy-nine participants completed the 16-item version of the prodromal questionnaire (PQ-16) and a 9-item questionnaire of perceptual and cognitive aberrations (PCA) for the assessment of basic symptoms (BS) online. 52.3% of participants met a priori cut-off criteria for the PQ and 73.6% for PCA items online. One thousand seven hundred eighty-seven participants were invited for a clinical interview and *n* = 356 interviews were conducted (response rate: 19.9%) using the Comprehensive Assessment of At-Risk Mental State (CAARMS) and the Schizophrenia Proneness Interview, Adult Version (SPI-A). *n* = 101 CHR participants and *n* = 8 first-episode psychosis (FEP) were detected. ROC curve analysis revealed good to moderate sensitivity and specificity for predicting CHR status based on online results for both UHR and BS criteria (sensitivity/specificity: PQ-16 = 82%/46%; PCA = 94%/12%). Selection of a subset of 10 items from both PQ-16 and PCA lead to an improved of specificity of 57% while only marginally affecting sensitivity (81%). CHR participants were characterized by similar levels of functioning and neurocognitive deficits as clinically identified CHR groups.

**Conclusion:**

These data provide evidence for the possibility to identify CHR participants through population-based web screening. This could be an important strategy for early intervention and diagnosis of psychotic disorders.

## Background

The identification of young people at high-risk for the development of severe mental health disorders is an important objective in current research and clinical practice.^1–4^ Over the last 3 decades, clinical criteria have been developed that allow the identification of participants with a high-risk of developing psychotic disorders, such as schizophrenia (ScZ).^5,6^ These include ultra high-risk (UHR) criteria that involve the presence of attenuated, psychotic symptoms as assessed by the Comprehensive Assessment of At-Risk Mental State (CAARMS) instrument^6^ or through the Structured Interview for Psychosis-Risk Syndromes (SIPS).^7^ Moreover, UHR criteria instruments include a genetic risk plus functional deterioration syndrome as well as brief limited intermittent psychotic episodes (BLIPs). In parallel, clinical high-risk (CHR) criteria also have been based on the basic symptom (BS) concept^8^ that are based on the presence of self-experienced perceptual and cognitive anomalies that are thought to represent the earliest manifestation of psychosis risk.^8^

There is now consistent evidence that these instruments allow a reliable identification of CHR participants^9^ with transition rates ranging between 10% and 50% over a 2- to 5-year period.^10^ Interestingly, recent studies have shown that the combined presence of both BS and UHR criteria increases the predictive power significantly.^11^ The importance of detecting CHR individuals is also underlined by the fact that at-risk populations show high correlations with nonpsychotic symptoms^12^ and mental health outcomes in those cases that do not develop psychotic disorders meet criteria for a range of other diagnoses, including affective disorders, personality disorders, and substance abuse.^13,14^

A major limitation for current approaches to detect CHR participants is the fact that CHR criteria need to be established through semi-structured interviews administered by trained personnel in help-seeking populations who are already within the health care system. Accordingly, it would be important to develop novel ways of identifying CHR participants that could potentially circumvent clinical entry points and allow population-wide screening of potential signs of impending psychosis and related mental disorders.

E-mental health applications, such as online screening for emerging mental health conditions, could provide an important approach to target young people at-risk for psychosis as they could facilitate identifying potential participants who would benefit from more detailed psychiatric assessments. To investigate this possibility, the Youth Mental Health Risk and Resilience Study (YouR Study) implemented a website http://www.your-study.org.uk which includes an initial screening through the 16-item version of the prodromal questionnaire (PQ-16)^15^ and a 9-item scale of perceptual and cognitive aberrations (PCA). Previous studies using questionnaire-based screenings in mental health settings and the general population highlighted the potential utility of using screening tools to identify CHR individuals.^16–18^ For example, the PQ-16 produced correct classification of UHR individuals in a secondary mental health setting with high sensitivity and specificity.^15^

Accordingly, we tried to determine whether questionnaire-based assessments of both attenuated psychotic experiences and BS through an online platform are characterized by sufficient sensitivity and specificity to predict CHR status as determined by clinical interviews. A secondary objective was to characterize the neurocognitive and psychopathological characteristics of CHR participants recruited through an online approach and compare results with at-risk samples in previous studies that were identified through clinical pathways.

Specifically, we predicted that self-reported levels of attenuated psychotic experiences and BS obtained online would predict CHR criteria as assessed through the CAARMS and SPI-A interviews. In addition, CHR participants identified through online screening were expected to be characterized by similar levels of functioning and neurocognitive deficits as clinically identified CHR individuals, suggesting that E-mental health applications may be effective in the detection of early signs of psychosis in the general population and in help-seeking participants.

## Methods

### Recruitment and Participants

The YouR Study is a cross-sectional study to identify neurobiological mechanisms and predictors of psychosis-risk with a state-of-the-art neuroimaging approach (magnetoencephalography, magnetic resonance spectroscopy, magnetic resonance imaging) in combination with core psychological processes, such as affect regulation, adverse experiences, and attachment.^19^ The YouR study is funded by the Medical Research Council (MRC).

A website was implemented http://www.your-study.org.uk as part of the study protocol and potential participants were invited through e-mail invitations, flyers, advertisements, and general practioners (GP) letters. Specifically, email invitations were sent out to colleges and Universities in Glasgow and Edinburgh. In addition, posters and flyers were advertised in NHS clinics and public transportation. Finally, databases of GP practices were searched for potential participants who then received an invitation letter that directed them to the website.

The study was approved by the ethical committees of the NHS Research Ethical Committee Glasgow & Greater Clyde.

Informed consent for the web screening was provided online, followed by 2 questionnaires: (*a*) the PQ-16^15^ and (*b*) a 9-item PCA scale that was developed to assess BS (Appendix). The PQ-16 was developed by Ising et al.^15^ from the 92-item prodromal questionnaire (PQ)^20^ in a help-seeking population attending secondary mental health services. A cut-off score of 6 or more items was associated with UHR criteria as assessed by the CAARMS with high sensitivity (87%) and specificity (87%). Items for the PCA were generated from existing patient descriptions of cognitive and perceptual experiences^21^ and from the SPI-A.^22^ Participants were asked to provide ratings based on experience in the last 12 months.

Cut-off criteria for further clinical assessments were 6 or more positively endorsed items on the PQ-16 based on previous data suggesting correct classification of CHR criteria based on CAARMS interviews with high sensitivity and specificity.^15^ For the PCA, a cut-off score of 3 or more positively endorsed items was selected.

### Clinical Assessments and CHR Criteria

**Fig. 1.**
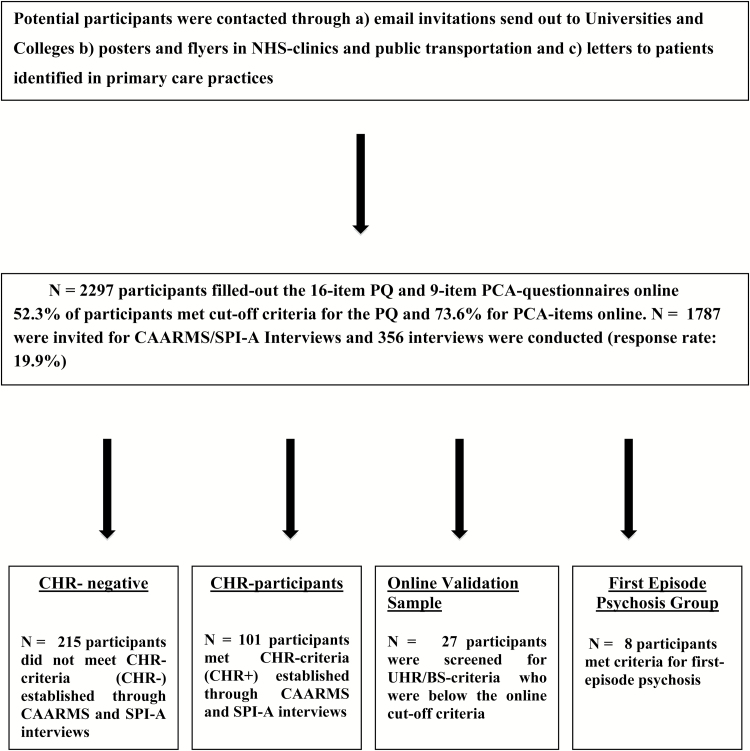
Recruitment and screening pathway.

Participants who met online inclusion criteria (PQ: ≥6 items; PCA: ≥3 items) were invited through an email to participate in the second part of the study that involved clinical assessments to determine CHR status and neuropsychology assessments (figure 1). At the beginning of the appointment, informed consent was obtained and the positive scale of the CAARMS^6^ and items of the Schizophrenia Proneness Instrument (SPI-A)^22^ to determine at-risk criteria as defined by cognitive-perceptive basic symptoms (COPER) and cognitive disturbances (COGDIS) were administered through trained research assistants and MSc/PhD level researchers. CHR participants were excluded for current or past diagnosis with Axis I psychotic disorders. Other co-morbid Axis I diagnoses, such as mood or anxiety disorders, were not exclusionary and all participants were between 16 and 35 years of age (for more details, see Uhlhaas et al^19^).

A second appointment served to verify CAARMS and SPI-A items and the M.I.N.I. International Neuropsychiatric Interview (M.I.N.I. 6.0).^19^ Moreover, the scales for premorbid adjustment,^23^ social and functional role scales^24^ and the Brief Assessment of Cognition in Schizophrenia Battery (BACS) were administered.^25^ Participants were recruited into the CHR group if they meet (*a*) SPI-A COGDIS/COPER criteria, (*b*) ARMS attenuated psychosis group (subthreshold psychotic syndrome present in the last year without a decline in functioning), (*c*) ARMS vulnerability group (family history of psychosis plus a 30% drop in the Global Assessment of Functioning [GAF] scale), and (*d*) ARMS BLIPs group (brief limited intermittent psychotic symptoms).

### Statistical Analyses

Data were analyzed using SPSS version 18.0.

### ROC Analyses

To investigate the sensitivity and specificity of online screening scores toward predicting CHR status, we conducted ROC analyses. Pairwise comparison of ROC curves was performed to test the statistical significance of the difference between the areas under the ROC curves of the PQ-16 and the PCA. The accuracy of the test depends on how well the test separates the group being tested into those with and without the disorder in question and is measured by the area under the curve (AUC). An AUC test value equal to 0.5 means that there is no difference and when the value equals 1 there is perfect separation of the values of the 2 groups.

## Results

### Screening Results

A total of *n* = 2296 participants completed the online screening from the commencement of the study in September 2014 until July 2017. 52.3% participants (*n* = 1202) fulfilled the PQ-16 cut-off criteria (≥6 or more items) while 73.6% (*n* = 1691) met criteria for the PCA (≥3 or more items). Participants were invited for interviews until July 2017 and 356 interviews were conducted (a response rate of 19.9%). There were no significant differences between participants who accepted invitations for interviews vs those who did not on the PQ-16 [*t*(1785) = 0.63, *P* = .53]. A statistical trend emerged for higher scores on the PCA scale for participants who attended clinical assessments [*t*(1785) = 1.81, *P* = .07].

### Sample Characteristics

CAARMS/SPI-A interviews were conducted and *n* = 101 participants met CHR criteria and *n* = 8 first-episode (FEP) criteria (mean age: 21.8 years; gender: 6 male; years of education: 13.67 years, ScZ: *n* = 3, schizoaffective disorder: 2: psychotic disorder not otherwise specified (NOS): 1, bipolar disorder with psychotic features: 1). FEP was defined as a person who fulfilled DSM-V for psychotic disorders that had previously not been in contact with mental health services. *N* = 215 were below CHR criteria (CHR negative group). For *n* = 32 participants, there was insufficient data to determine CHR status due to withdrawal from the interview and/or the tattoos/piercings that were exclusion criteria of the neuroimaging protocol. These participants were excluded from subsequent analysis. For 14 CHR participants, neuropsychological data were not available. Of the 101 CHR participants, *n* = 87 were recruited from the general population (e-mail invitations, flyers, and posters) while 14 were recruited through the National Health Service (NHS) (GP letters, posters).

In addition, we carried out SPI-A and CAAMRs interviews in *n* = 27 participants who scored below online cut-offs for PQ and PC-Q items (online validation sample, OVS). Two out of 27 participants met CHR criteria. However, there was a significant delay between the initial online score and clinical assessments, suggesting that CHR symptoms emerged after the entry of questionnaire data.

Demographic and clinical characteristics of CHR participants, CHR negative, OVS, and control groups as well as a sample of matched control participants are summarized in table 1. Fourteen CHR participants were in current treatment for mental health problems (NHS services: *n* = 8; other services (student counseling, charities): *n* = 7). Thirty-one CHR participants disclosed no previous or current contact with mental health services while 55 had previous contact (*n* = 16 NHS, *n* = 13 other services). For 10 CHR participants, no information was available.

**Table 1.** Demographical and Clinical Characteristics of Participants

	CHR Group (*N* = 101)	CHR Negative (*N* = 215)	OVS (*N* = 27)	Controls (*N* = 38)	Statistics^a^
Mean age (y)	22.03 (4.0)	22.71 (4.6)	19.81 (2.1)	23.07	.05 (CHR vs OVS)
Gender (male/female)	24/77	69/146	5/22	16/26	.05 (CHR vs OVS)
Education (y)	15.74 (2.9)	15.74 (3.1)	14.56 (2.3)	16.63 (2.8)	.05 (CT vs OVS)
CHR category					
SPI-A	71				
CAARMS	73				
SPI-A/CAARMS	43				
BACS data^b^					
Verbal memory	50.84 (11.0)			50.42 (8.9)	.83
Digit sequencing	20.72 (4.0)			20.66 (2.7)	.93
Token motor task	67.83 (14.1)			79.03 (12.9)	.01
Verbal fluency	57.06 (13.9)			59.45 (14.6)	.39
Symbol coding	67.78 (13.3)			74.42 (9.8)	.05
Tower of London	18.21 (2.4)			18.37 (1.9)	.72
Total score	283.34 (36.0)			302.34 (25.9)	.05
GAF	61.06 (12.1)	75.32 (11.3)	76.75 (8.3)	88.68 (5.3)	.00 (CT vs CHR^+^, CT vs CHR^−^, CT vs OVS, OVS vs CHR^+^, OVS vs CHR^−^, CHR vs CHR^+^
Role/social functioning					
Role current	7.60 (1.1)			8.63 (0.7)	.01
Social current	7.77 (0.9)			8.89 (0.4)	.01
Premorbid adjustment					
Childhood	1.02 (0.9)			0.36 (0.6)	.01
Early adolescence	1.13 (0.8)			0.49 (0.6)	.01
Late adolescence	1.04 (0.7)			0.69 (0.9)	.01

^a^All statistical comparisons were carried out with a one-way ANOVA except for differences in gender where a chi-squared test was applied. Standard deviations are in brackets where appropriate.

^b^BACS data was only available from *n* = 87 CHR participants.

CHR participants were characterized by a significant lower GAF score compared with CHR negative participants. The OVS group was significantly younger than CHR negative participants and had fewer male participants and had less years of education.

Compared with controls, the CHR group was characterized by reduced role and social functioning as well as impaired premorbid adjustment. The large majority of CHR participants met also criteria for Axis-1 affective and anxiety disorders (table 2). Comparisons on neuropsychological measures revealed that CHR participants showed significant deficits in motor speed, symbol coding, and in the BACS composite score compared with controls (table 1).

**Table 2.** Clinical Characteristics of CHR^+^ Participants

Diagnosis	
Anxiety disorder	77.8%
Mood disorder	61.1%
Post-traumatic stress disorder	9.0%
Eating disorder	13.8%
Learning disability	
a) Autism	1%
b) ADHD	3%
c) Dylexia	8%
Medication	
Anti-depressants	17.9%
Anti-psychotics	0%
Anxiolytics	0%
Other	11%

### Internal Consistency of PCA

Cronbach’s alpha was measured to assess the internal consistency of the PCA questionnaire. The total score on PCA was associated with a value of 0.786, suggesting that the items have high internal consistency.

### ROC Analyses

**Fig. 2.**
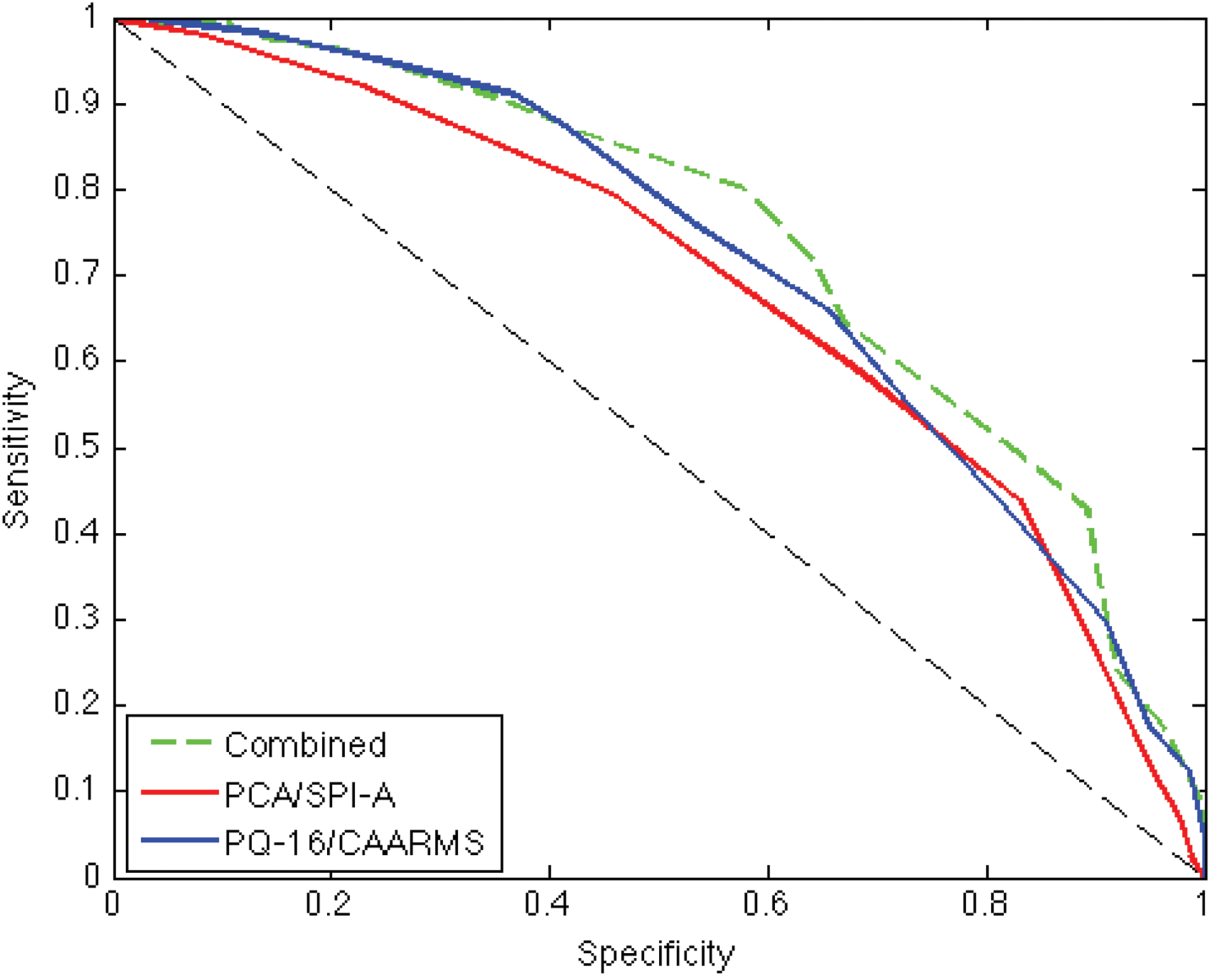
Receiver operating characteristic (ROC) curves of predictive values for online questionnaires scores toward CHR status. Blue line: ROC curve for PQ-16 scores in relationship to UHR status as assessed by the CAARMS. Red line: ROC curve for PCA scores in relationship to SPI-A CHR status. Green line: ROC curve for combined PQ-16/PCA scores and CHR status. Black dashed line: reference line of performance no better than chance.

ROC curves were plotted and AUC calculated to assess the predictive value of online PQ-16 and PCA scores in relationship to CHR status for interviewed participants that included *n* = 27 participants that did not meet online cut-off criteria (total sample: *n* = 351) (table 3, figure 2).

**Table 3.** ROC-Analyses for PQ-16, PCA and PCA/PQ-16 Combined

Measure	Cut-off	Sensitivity	Specificity	PPV %	NPV %	LR^+^	AUC	Standard Error	95% CI	*P*
PQ-16	6	0.81	0.44	29	89	1.45	0.72	0.033	0.66–0.78	<.001
PQ-16	7	0.73	0.55	32	88	1.62				
PCA	3	0.95	0.13	25	89	1.06	0.69	0.033	0.62–0.75	<.001
PCA	4	0.90	0.26	27	90	1.22				
PCA	5	0.83	0.44	31	89	1.48				
Combined	10	0.89	0.43	42	89	1.56	0.74	0.028	0.69–0.80	<.001

Note: PPV is the positive predictive—true positive/(true positive + false positives); NPV is the negative predictive value—true negative/(true negative + false negative). The NPV is very high for all these measures ie, a negative score really is likely to be negative. The PPV is relatively low (above threshold is 1/3 chance of being a genuine positive).

The AUC for the PQ-16 for predicting CAAMRS status was significant indicating that total symptom endorsement score was predictive of CAARMS status above chance level. A cut-off score of 6 or more symptom items on the PQ-16 was found to identify at-risk status with a sensitivity of 81% and specificity of 44%. Increasing the cut-off score to 7 endorsed items decreased sensitivity to 73% but increased specificity 55%.

The AUC for the PCA was also significant (figure 2). A cut-off score of 3 or more symptom items endorsed was found to identify SPI-A status with a sensitivity of 95% and specificity of 13%. An alternative threshold of 4 and 5 items or more symptom items endorsed improved specificity (4 items: sensitivity = 90%; specificity: 26%; 5 items: sensitivity = 83%; specificity: 44%) (table 3).

To assess the utility of combining the subscales of the online questionnaire to predict CHR status further ROC analysis was conducted where the PQ-16 and PCA subscales were combined (table 3, figure 2). The AUC was significant, indicating that symptom endorsement score across the subscales was predictive of CHR status. A cut-off score of 10 or more symptom items endorsed was found to identify CHR status with a sensitivity of 89% and specificity of 43%, thus slightly improving scores from the individual measures.

To examine the possibility to improve the specificity of the online screener, we implemented a machine-learning approach implemented in MATLAB 2015a: RELIEFF^26^ which estimates the utility of features to a classification problem in the situation where features may have conditional dependencies. For this analysis, we selected all 25 items from both the PQ-16 and the PCA in addition to 3 demographical variables: (*a*) age, (*b*) sex, and (*c*) familial risk (first degree relative with a psychotic disorder) that were obtained during the initial clinical assessment. We took the average predictive weights from 10 samples of 234 individuals’ data set and ranked the features (supplementary figure 1; table 4). The presence of a family member was ranked the seventh most predictive, while neither age, nor gender proved more useful than any of the questionnaire responses. We then recalculated the ROC curves, adding familial risk to the original 25 features.

**Table 4.** ROC Analyses for a Subset of Questionnaire and Demographic Data

Number of Items	Threshold	Sensitivity	Specificity	PPV %	NPV %	LR^+^	AUC	SE	95% CI	*P*
12	5	0.80	0.57	46	86	1.86	0.73	0.028	0.67–0.78	<.001
11	4	0.84	0.51	45	77	1.71	0.72	0.03	0.65–0.77	<.001
10	4	0.81	0.57	54	77	1.88	0.71	0.028	0.65–0.76	<.001

*Note:* PPV is the positive predictive—true positive/(true positive+ false positives); NPV is the negative predictive value—true negative/(true negative +false negative). The NPV is very high for all these measures, ie, a negative score really is likely to be negative. The PPV is relatively low (above threshold is 1/3 chance of being a genuine positive).

We then recomputed the ROC curve for increasing numbers of features, in sequence, adding one feature at a time, in order of predictive rank determined by RELIEFF. We then compared the specificity of solutions with the sensitivity value closest to 80%. We found that a smaller number of items (10 or 12) that optimized the likelihood ratio (LR+) that included familial risk, improved specificity (57%), while only marginally affecting sensitivity (81%).

## Discussion

The current study examined the possibility of detecting CHR participants through a web-based screening approach that utilized the PQ-16 as well as a 9-item PCA scale in the general population and through clinical services. Our data suggest that both screening instruments are characterized by good to moderate sensitivity and specificity to predict CHR status as defined by CAARMS and SPI-A interviews.

The PQ and abbreviated versions have been used in previous studies to detect CHR participants in secondary mental health settings.^15,18,27^ The online implementation of the PQ-16 in combination with a previously established cut-off 6 or more items yielded similar sensitivity and specificity values to predict UHR status established through a semi-structured interview as in the study by Ising et al.^15^ Specifically, we observed that a score of 6 or more positively answered items was associated with UHR status as assessed by the CAARMS with good sensitivity (81%) and specificity (44%).

A novel approach of the current study was the implementation of a screening instrument to predict self-experienced PCAs that are highly predictive for later transitioning to psychosis.^5,11^ Currently, it is unclear whether BS can be reliably predicted from self-report measures. The 9-item PCA was characterized by excellent sensitivity for predicting COPER and COGDIS status assessed through the SPI-A interview (95%) but low specificity (13%). Our data show that changing the cut-off to 4 or more items was associated with improved specificity.

The combination of a subset of features from the PQ-16 and PCA that included familial risk furthermore improved specificity (57%), highlighting the potential for improved online screening using a novel combination of features. However, we wish to note that this screener would require further validation in an independent sample.

An important issue concerns the clinical and demographic characteristics of CHR participants in the current sample who are self-referred and identified through an online screening from the general population and clinical services. Overall, the majority of CHR participants were recruited from the general population and only a minority were in current or past treatment with NHS services. Our data indicate that the current sample shares similarities with existing CHR populations identified through clinical referral pathways. Specifically, the CHR participants identified through our web interface are characterized by reduced GAF scores and impaired role and social functioning. However, especially role and social functioning was somewhat higher than in previous CHR samples.^24,28^

Consistent with previous data,^29^ we observed mild impairments in neurocognition in CHR participants compared with healthy controls. Specifically, reductions on the digit symbol and motor token task were observed that replicate findings highlighting the contribution of reductions in processing speed toward psychosis risk.^30,31^ Interestingly, both verbal memory and executive functions were found largely to be intact in the current CHR sample that is in contrast to deficits reported in previous studies.^29,32,33^

Baseline assessment of current psychopathology furthermore revealed extensive psychiatric co-morbidity of CHR participants. In particular, both anxiety and mood disorders were present in the large majority of CHR participants consistent with previous formulations that that changes in affect are frequent early signs before the onset of psychosis.^34^ Together with the presence of PTSD and eating disorders in the current sample, these findings support the view that CHR participants express a high degree of comorbidity with other psychiatric syndromes^12–14^ which are likely to impact on the trajectory and functional outcome.^35^

### Limitations and Further Steps

The current findings have a number of limitations. Firstly, we could only verify at-risk criteria established through online screening for UHR and BS symptoms in a subgroup of individuals as only 19.9% of those participants who met online screening cut-offs were evaluated through CAARMS and SPI-A interviews. However, the current data suggest that there were no significant differences in scores on the PQ-16 and only a trend for difference PCA scores. Secondly, we could only establish in a small sample of *n* = 27 participants, the relationship between scores below the online cut-off for CHR status and SPI-A as well as CAARMS criteria. Finally, since both PQ-16 and PCA measures were completed online, it is possible there was an overendorsement of PLE and BS (see de Jong et al^36^ for a recent study in adolescents). Moreover, it was not possible to determine how many of the target population visited our website. However, we would like to note that the frequency of both PLEs as well as CHR criteria were significantly less prevalent in our online sample compared to estimates obtained from the general population,^37,38^ suggesting that the help-seeking participants were self-selecting with significantly higher levels of PLE as well as affective disorders.

In the current sample, ~1 out of 3 participants who met online cut-off criteria was also found to either meet UHR or SPI-A status, suggesting that population wide-screening may be useful in the detection of emerging signs of psychosis and related mental health outcomes. In addition, we also would like to note that the current recruitment approach identified a significant number of participants meeting criteria for FEP, further highlighting the potential of an online screening for identifying young people that are at-risk for serious mental health problems.

However, several issues need to be addressed in future studies to improve the clinical utility of an E-mental health approach to detect emerging psychosis. Firstly, follow-up data on the current sample will provide further information on transition rates of CHR participants identified through online screening. There is emerging evidence that at-risk samples recruited primarily through outreach activities may be characterized by lower transition rates than in samples referred through clinical pathways.^10,39^ Together with the modest specificity of our online screening, this raises the issue of a substantial number of false positive and the risk of conferring stigma in detecting CHR participants.^40^ We would like to note, however, that in the course of interviewing a large number of CHR participants recruited through our online pathway, we rarely encountered concerns about being labeled. This anecdotal observation is consistent with data suggesting that young people who are deemed to be at potential risk for psychosis value the opportunity of talking about their experiences rather than express explicit concerns about stigma.^41^

Nonetheless, it is important that future studies investigate the possibility of improving sensitivity and specificity of online detection of CHR participants through additional variables, such as premorbid adjustment, psychosocial functioning and neurocognition, that are associated with the CHR status and psychosis risk.^42,43^ This is supported by emerging evidence suggests that additional clinical and demographic variables increase accuracy of predicting conversion to psychosis in clinically ascertained CHR samples.^42,43^ Such refined online screening could also potentially allow the detection of FEP cases as demonstrated by findings from the PQ-16 in our sample that showed that the FEP group had significantly elevated scores compared to CHR participants. This issue is potentially important as current evidence suggests that specialized high-risk clinics only identify around 5% of FEP cases.^42^ Accordingly, development of novel tools that allow the identification of both FEP cases as well as participants meeting CHR criteria would be important for early intervention.

## Summary

In summary, our data provide evidence that it is possible to identify CHR participants through online screening at the population level. This approach could have implications for efforts to identify young people at-risk of mental health problems as well as for the identification of emerging psychosis. Future studies are required to improve sensitivity and specificity through the addition of clinical, demographic, and neurocognitive variables. Moreover, it is conceivable that the current framework could be extended to secondary mental health settings as well as for related disorders that typically emerge during the transition from adolescence to adulthood, such as affective disorders and borderline personality disorder.

## Funding

This study was supported by the project MR/L011689/1 from the Medical Research Council (MRC).

## Supplementary Material

Supplementary Figure 1

## Appendix:

### PCA Questionnaire

Instructions: You are asked to complete a questionnaire which will ask about recent thoughts, feelings, and experiences. We are interested in exploring the links between the presence of thoughts and feelings and the potential risk for the development of mental health problems.

Please answer the questions honestly based *only* on how you have been feeling in *the last 12 months*.

Please note that you should *not* answer questions based on recent experiences that occurred only while under the influence of alcohol, drugs, or medications that were not prescribed to you.

**Table:**

		If true: how much distress did you experience?					
		True	False	None	Mild	Moderate	Severe
1.	Do you have sometimes the impression that you cannot understand spoken or written words immediately, although these words are familiar to you?						
2.	Have you noticed that you cannot remember familiar words or terms so that you have to search for them longer or that you have to use other words instead?						
3.	Does it happen sometimes that too many thoughts race through your head without any connection between them?						
4.	Do you sometimes find it more difficult than in the past to direct your attention onto 2 different things, eg, to follow a conversation and at the same time to take some notes, to drive a car or wash dishes?						
5.	Does it happen to you that you can only concentrate with difficulties on a movie or a book, because nonrelevant thoughts distract or bother you, although these topics are actually without importance to you?						
6.	Have you recently experienced that your stream of thoughts is getting disrupted, so that your present thought disappears or you cannot follow it anymore?						
7.	Does it happen to you that experiences or conversations go through your mind again and again although these are actually nonsignificant and you would prefer to occupy yourself with other things?						
8.	Does it happen to you that you can only hear snatches of conversation but you cannot fit them together in a meaningful way?						
9.	Do things sometimes appear to be split up like a photograph that’s torn in bits and put together again?						
