# Supplementary material for: Using Online Screening in the General Population to Detect Participants at Clinical High-Risk for Psychosis
Source: Schizophr Bull. 2018 Jun 8;45(3):600–9. doi: 10.1093/schbul/sby069 (PMC6483579; doi:10.1093/schbul/sby069)
Supplement: Supplementary Figure 1 [file sby069_suppl_supplementary_material.doc]

**Supplementary Material Figure 1.**

**
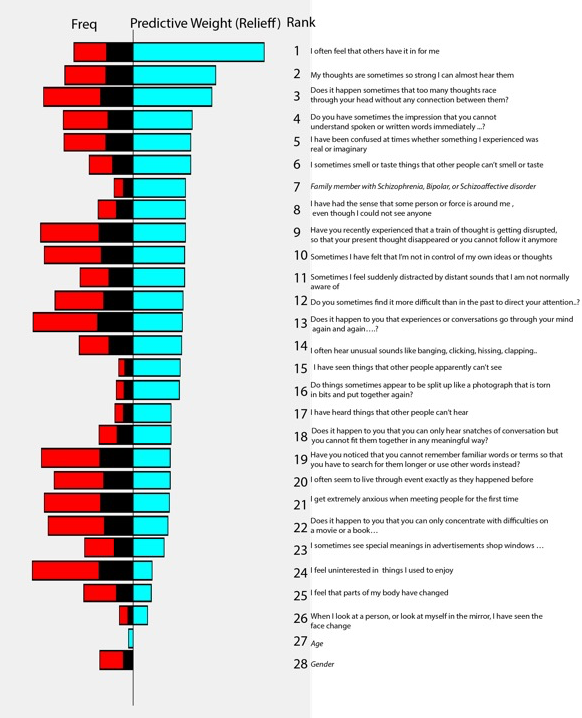
**

Figure 1. The blue bars show the relative magnitude of the predictive weight calculated by the Relieff algorithm shown in order of rank, neighbours = 234. Weights were averaged over 10 splits of the data. Red bars show the relative frequency of endorsement of each item in the interviewed set (352), and the black shows the relative number of endorsements in the CAARM+, SPIA + groups in the entire data set. On the Right side, we show the questions (in order of predictive rank. Items 7,27, and 28, are demographic items, in italics.
